# *RUNX1-PDCD6* fusion resulting from a novel t(5;21)(p15;q22) chromosome translocation in myelodysplastic syndrome secondary to chronic lymphocytic leukemia

**DOI:** 10.1371/journal.pone.0196181

**Published:** 2018-04-19

**Authors:** Ioannis Panagopoulos, Ludmila Gorunova, Eva-Marie Jacobsen, Kristin Andersen, Francesca Micci, Sverre Heim

**Affiliations:** 1 Section for Cancer Cytogenetics, Institute for Cancer Genetics and Informatics, The Norwegian Radium Hospital, Oslo University Hospital, Oslo, Norway; 2 Department of Haematology, Oslo University Hospital, Oslo, Norway; 3 Institute of Clinical Medicine, Faculty of Medicine, University of Oslo, Oslo, Norway; European Institute of Oncology, ITALY

## Abstract

Leukemic cells often carry chromosome aberrations which generate chimeric genes of pathogenetic, diagnostic, and prognostic importance. New rearrangements giving rise to novel fusion genes define hitherto unrecognized genetic leukemia subgroups. G-banding, fluorescence in situ hybridization (FISH), and molecular genetic analyses were done on bone marrow cells from a patient with chronic lymphocytic leukemia (CLL) and secondary myelodysplasia. The G-banding analysis revealed the karyotype 46,XX,del(21)(q22)[9]/46,XX[2]. FISH on metaphase spreads with a *RUNX1* break apart probe demonstrated that part of *RUNX1* (from 21q22) had moved to chromosome band 5p15. RNA sequencing showed in-frame fusion of *RUNX1* with *PDCD6* (from 5p15), something that was verified by RT-PCR together with Sanger sequencing. Further FISH analyses with *PDCD6* and *RUNX1* home-made break apart/double fusion probes showed a red signal (*PDCD6*) on chromosome 5, a green signal on chromosome 21 (*RUNX1*), and two yellow fusion signals, one on der(5) and the other on der(21). Reassessment of the G-banding preparations in light of the FISH and RNA-sequencing data thus yielded the karyotype 46,XX,t(5;21)(p15;q22)[9]/46,XX[2]. The t(5;21)(p15;q22)/*RUNX1-PDCD6* was detected only by performing molecular studies of the leukemic cells, but should be sought after also in other leukemic/myelodysplastic cases with del(21q).

## Introduction

Microscopic studies of hematologic malignancies, including myelodysplastic syndrome (MDS), acute myeloid leukemia (AML), and acute lymphoblastic leukemia (ALL), have shown that they often carry acquired chromosomal changes which generate chimeric genes of pathogenetic, diagnostic, and prognostic importance [1].

In the early 90s, work on the molecular characterization of the translocation t(8;21)(q22;q22) in AML led to identification of the *RUNX1* gene (previously called *AML1*, *CBFA2*, *PEBP2aB*) from the 21q22 breakpoint and its fusion with *RUNX1T1* (previously called *AML1-MTG8*, *AML1T1*, *CBFA2T1*, *CDR*, *ETO*, *MTG8*, *ZMYND2*) from 8q22 [2]. *RUNX1* codes for the alpha subunit of the core binding factor (CBF) and plays an important role in normal hematopoiesis [2, 3]. Genetic aberrations resulting in rearrangement of *RUNX1*, including generation of *RUNX1* fusion genes, have been shown to be critical events in both myeloid and lymphoblastic acute leukemias [2, 3]. Currently, the *RUNX1* gene has been reported to fuse with more than 40 different partner genes coding for structurally diverse proteins [1]. Some of the *RUNX1*-fusions are common, such as *ETV6-RUNX1*/t(12;21)(p13;q22) in pre-B-ALL and *RUNX1-RUNX1T1*/t(8;21)(q22;q22) in AML; they have been extensively studied and their prognostic impact is known [4, 5]. Others have been reported in only few or single cases and their possible prognostic impact remains unknown [1].

It is of clinical as well as scientific interest to report new chromosomal aberrations that give rise to novel fusion genes in hematologic malignancies. The t(7;21)(p22;q22) which generates the *RUNX1-USP42* fusion gene was originally found in a 7-year-old boy with AML-M0 [6]. Today, t(7;21)(p22;q22)/*RUNX1-USP42* is considered a rare nonrandom genomic aberration of myeloid malignancies which is frequently seen together with del(5q) [6–9].

We here present the genetic and clinical features of a case of chronic lymphocytic leukemia (CLL) with secondary myelodysplasia in which a novel t(5;21)(p15;q22) chromosome translocation was found that led to the formation of a *RUNX1-PDCD6* fusion gene.

## Materials and methods

### Ethics statement

The study was approved by the Regional Committee for Medical and Health Research Ethics, South-East Norway (S-07474a, REK Sør-Øst; http://helseforskning.etikkom.no) and written informed consent was obtained from the patient to publication of the case details prior to her death. The ethics committee’s approval included a review of the consent procedure. All patient information has been de-identified.

### Case report

A 57-year-old woman was diagnosed with CLL in 2004. The CLL cells expressed VH3-21 which showed 99% homology to germ line and were negative for CD38 and ZAP-70. Interphase FISH analyses showed no deletions of 6q23 (MYB), 11q22 (ATM), 13q14, or 17p13 (TP53), nor was there any indication of trisomy 12.

During the years 2005, to 2009 she received a total of 6 courses with fludarabine and 3 courses of fludarabine and cyclophosphamide. In 2015, four courses with benadamustine and rituximab were given with G-CSF support. A bone marrow assessment in October 2016 showed that dysplastic features involving erythropoiesis and thrombopoiesis had developed; there were now 5–10% CD34 positive cells whereas CLL cells accounted for 25–30% of the total. It was concluded that secondary myelodysplastic syndrome with excess of blasts had developed. From November 2017 she received treatment with the Bruton tyrosine kinase inhibitor ibrutinib. She died in February 2018 after having developed acute myeloid leukemia.

### G-banding and fluorescence in situ hybridization (FISH)

Bone marrow cells were cytogenetically investigated by standard methods at the time of diagnosis of secondary myelodysplastic syndrome as described in detail previously [9, 10].

Because the patient originally had CLL, interphase FISH analyses of bone marrow cells were performed using the Cytocell multiprobe CLL panel (Cytocell, http://www.cytocell.com) looking for deletions of *MYB* (6q23.3), *ATM* (11q22.3), *TP53* (17p13.1), and chromosome band 13q14.3, *IGH-BCL2* and *IGH-CCND1* fusions, *IGH* rearrangements in general, and extra copies of chromosome 12. After del(21)(q22) was found by karyotyping (see below), further FISH analyses were performed on metaphase spreads using the *AML1* (*RUNX1*) break apart probe (Cytocell).

After the identification of a *RUNX1-PDCD6* fusion transcript (see below), additional FISH analyses were again performed on metaphase spreads now using *RUNX1* and *PDCD6* home-made break apart/double fusion probes as described in detail previously [10]. For the *RUNX1*gene on chromosome 21, the BAC clones used were RP11-272A3 (Position: chr21:34474173–34659333; Band: 21q22.12; GRCh38/hg38 Assembly) and RP11-768B1618 (Position: chr21:35214983–35391484; Band: 21q22.12; GRCh38/hg38 Assembly). For the *PDCD6* gene on chromosome 5, the BAC clones used were RP11-811I15 (accession number AC113430, Position: chr5: chr5:49931–95412; Band: 5p15.33) and CTD-2228K2 (accession number AC010442, Position: chr5:370785–482715; Band: 5p15.33). The probes for *RUNX1* and *PDCD6* were labelled with Fluorescein-12-dCTP (PerkinElmer, Boston, MA, USA) and Texas Red-5-dCTP (PerkinElmer) in order to obtain green and red signals, respectively.

### RNA sequencing and PCR analysis

RNA sequencing was performed as described in detail previously [11]. Total RNA was extracted from the patient´s bone marrow cells at the time of secondary MDS diagnosis using miRNeasy Mini Kit (Qiagen Nordic, Oslo, Norway) and one μg was sent to the Genomics Core Facility at the Norwegian Radium Hospital, Oslo University Hospital (http://genomics.no/oslo/) for high-throughput paired-end RNAsequencing. The software FusionCatcher [12] was used to find fusion transcripts.

The procedures for reverse transcriptase-Polymerase Chain Reaction (RT-PCR) and direct sequencing of PCR products were previously described [13]. For amplification of *RUNX1- PDCD6* fusion transcript, two primer sets were used: 1) the forward RUNX1-809N-F1 (CGG CAG AAA CTA GAT GAT CAG ACC A) together with the reverse PDCD6-354R1 (TGA TGT ACT TCC ACA CAC CCG TGA) and 2) the forward RUNX1-852N-F1 (TTT CCG AGC GGC TCA GTG AAC) together with PDCD6-373R1 (GAC GTT CTG CCA GTC CGT GAT G). PCR cycling included initial denaturation at 94 °C for 30 sec followed by 35 cycles of 7 sec at 98 °C, 30 sec at 58 °C, and 30 sec at 72 °C with a final extension for 5 min at 72 °C.

## Results

### G-banding and FISH

**Fig 1:**
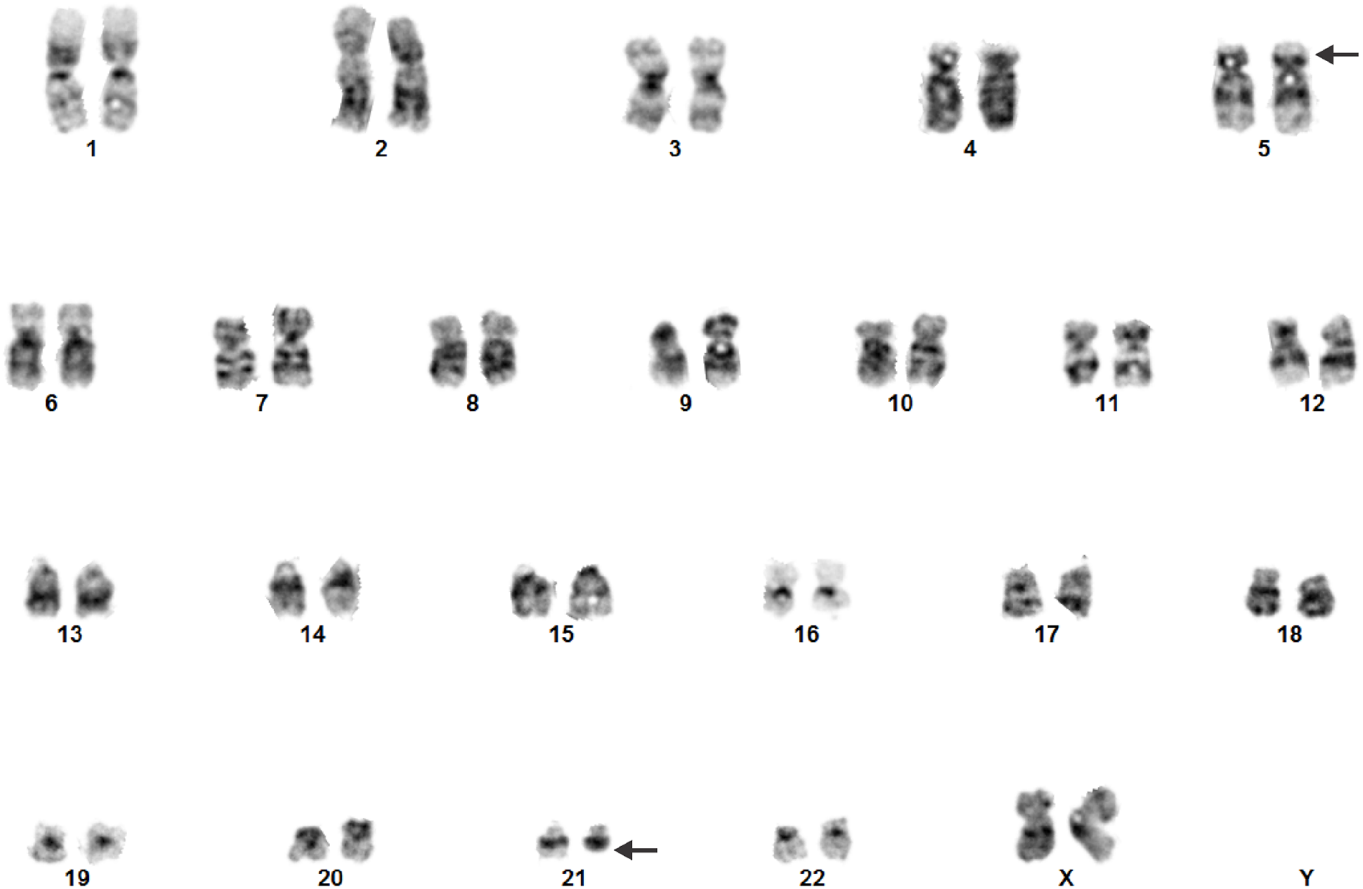
G-banding analysis of bone marrow from a patient with CLL and secondary MDS. The initial analysis showed a del(21)(q22). Reassessment of the G-banding preparations after taking into account additional molecular-cytogenetic data showed a t(5;21)(p15;q22). Arrows indicate breakpoints.

The initial G-banding analysis revealed the karyotype 46,XX,del(21)(q22)[9]/46,XX[2] (Fig 1).

**Fig 2:**
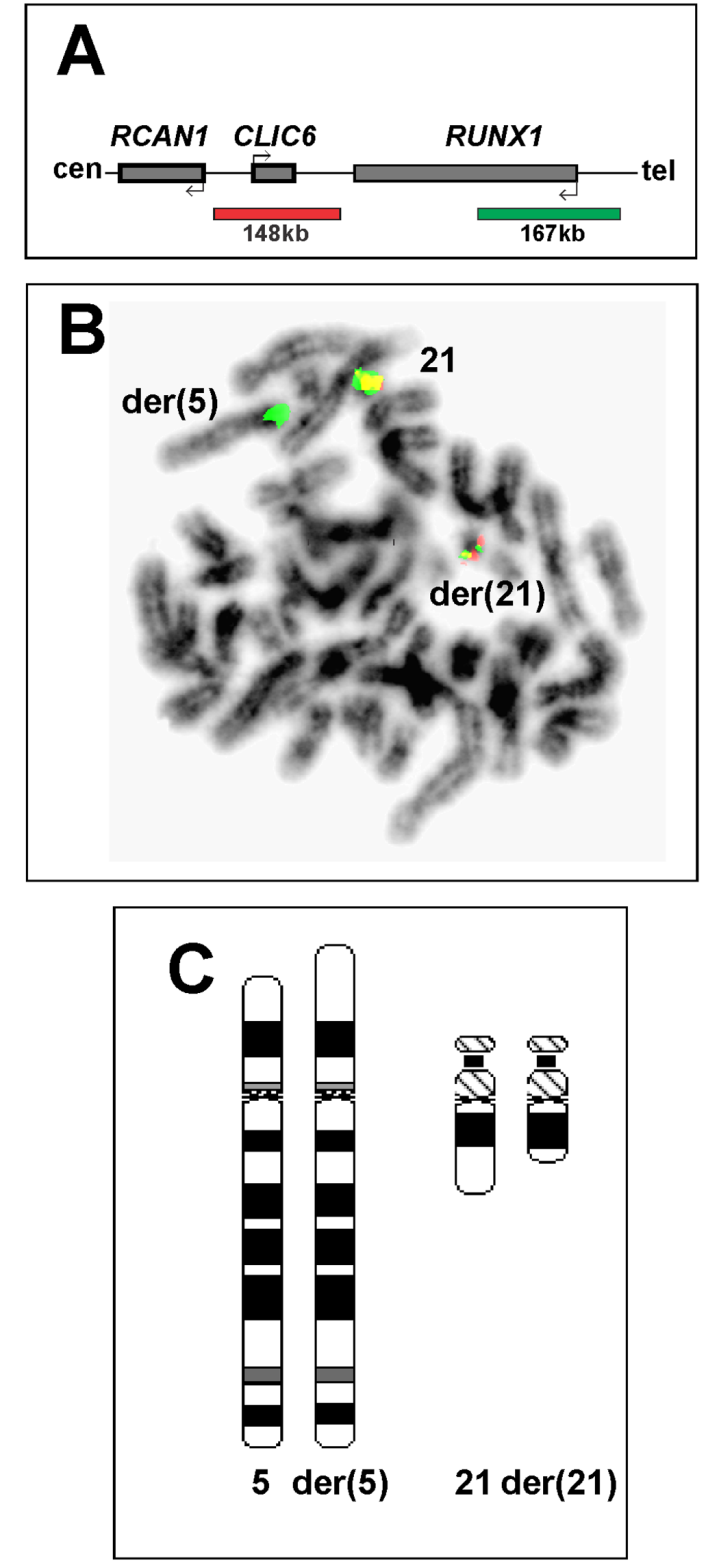
FISH analysis of bone marrow from a patient with CLL and secondary MDS using a commercial *RUNX1* break apart probe. (A) Diagram illustrating the commercial *RUNX1* probe. (B) Metaphase spread showing splitting of *RUNX1*. Most of the green signal has moved to distal 5p. (C) Ideograms showing the der(5)t(5;21)(p15;q22) and der(21)t(5;21)(p15;q22) together with their normal chromosome homologs.

FISH analysis with a multiprobe CLL panel did not detect loss of *MYB* (6q23), *ATM* (11q22), *TP53* (17p13), and chromosome band 13q14.3, nor was there any indication of *IGH-BCL2*, *IGH-CCND1* or other *IGH* splits or extra copies of chromosome 12 (data not shown). FISH with the commercial *RUNX1* break apart probe (Fig 2A) on metaphase spreads showed splitting of the *RUNX1* locus and that most of the green signal had unexpectedly moved to distal 5p (5p15; Fig 2B). The findings thus suggested the presence of a t(5;21)(p15;q22) chromosome translocation (Fig 2C).

### RNA-sequencing

Using the FusionCatcher software on the fastq files of the RNA sequencing data, 11 fusion genes were found (Table 1), among them a fusion of *RUNX1* with the *PDCD6* gene which maps on chromosome band 5p15.33 (https://www.ncbi.nlm.nih.gov/gene/10016).

**Table 1:** Fusion transcripts detected using FusionCatcher.

Gene_1 (5´-fusion partner)	Gene_2 (3´-fusion partner)	Fusion_sequence
*RUNX1*	*PDCD6*	CACTCCACTGCCTTTAACCCTCAGCCTCAGAGTCAGATGCAGG* GCACGTGGACTCCCTTTAATCCAGTGACTGTCAGGTCGATCAT
*AL122127*.*25*	*KIAA0125*	AGGGATCTGGGCATGAGGCCCCTTCTCCCAGGAGGGGAGGCAC* AGCCTGGAGACACAGGCCCCATCCTTCCCAATGGGGACACTTC
*GLYCTK*	*DNAH1*	ACTGGCTGAGGGACTCACAGCTGATGACCTGCTGCTCGTGCTGATCTCAG* TGATCACTGAGTACCTGTGTGAATGCCAACAAGAAAAATAAAACCACAGT
*TNFRSF17*	*SNX29*	CTGCCAGCTGCTTTGAGTGCTACGGAGATAGAGAAATCAATTTCTGCTAG* GATCACAGAACAATGACAAAAGACAATTTCTGCTGGAGCGACTGCTGGAT
*CHST11*	*MALAT1*	GTGTGGTGGTGCGTGCCTGTAGTCCCAGCTACTCGGGAGGCTGAGGTGGG* ACTTTTAGAAAGCTGTCTCCTTATTTAAATAAAATAGTGTTTGTCTGTAG
*MED12*	*IRF2BPL*	TCTTATAGCAGCAGCAGCAACAGCAACAGCAGCAGCAGCAGCAGCAGCAA*C AGCAGCAGCAGCAGCAGCAGCAGCAACAACAGCTCAACCACGTTGATGG
*METTL16*	*MNT*	ACTTCGACGTTCAGACCTGGGTTCCCGTCCAGTTCCATTCCTTGCACAGG* AGGAGCAGGAGCGGCTTCGCTTGGAGCAGGAGCGAGAGCAGGAACAGAAG
*STX16*	*NPEPL1*	TTGAACAGTCCTGTATCAAAACTGAAGATGGTTTGAAACAGCTTCACAAG* TGCCTGCTCTGTGCCAGGAACTGGAGGAAAAAAAAACAACAAAAAGTCAA
*TTN*	*ACTB*	CAAGTCATTTTATAATTGTTGTCCTTTGTTTTTTTTGTTTTGTTTTGTTT* TTTTTTTTTTTTTTTTGGCTTGACTCAGGATTTAAAAACTGGAACGGTGA
*ZER1*	*ZDHHC12*	TTAAGATGGCGACCGCACGGCAGGAGACCAAGGAAATGGCCCG* AGCTGCGGCAATGGGAGGAGCAGGGGGAGCTGCTCCTGCCCCT
*METTL23*	*MFSD11*	AGAATCCCAAGGTCCAATTGTGGTCTACTTATCAAGTTAGGAG* GAGACTTGGGGGGACTCTTCGATCGCGACCTGGATTTGGATTC

### Molecular and FISH confirmation of the *RUNX1-PDCD6* fusion

**Fig 3:**
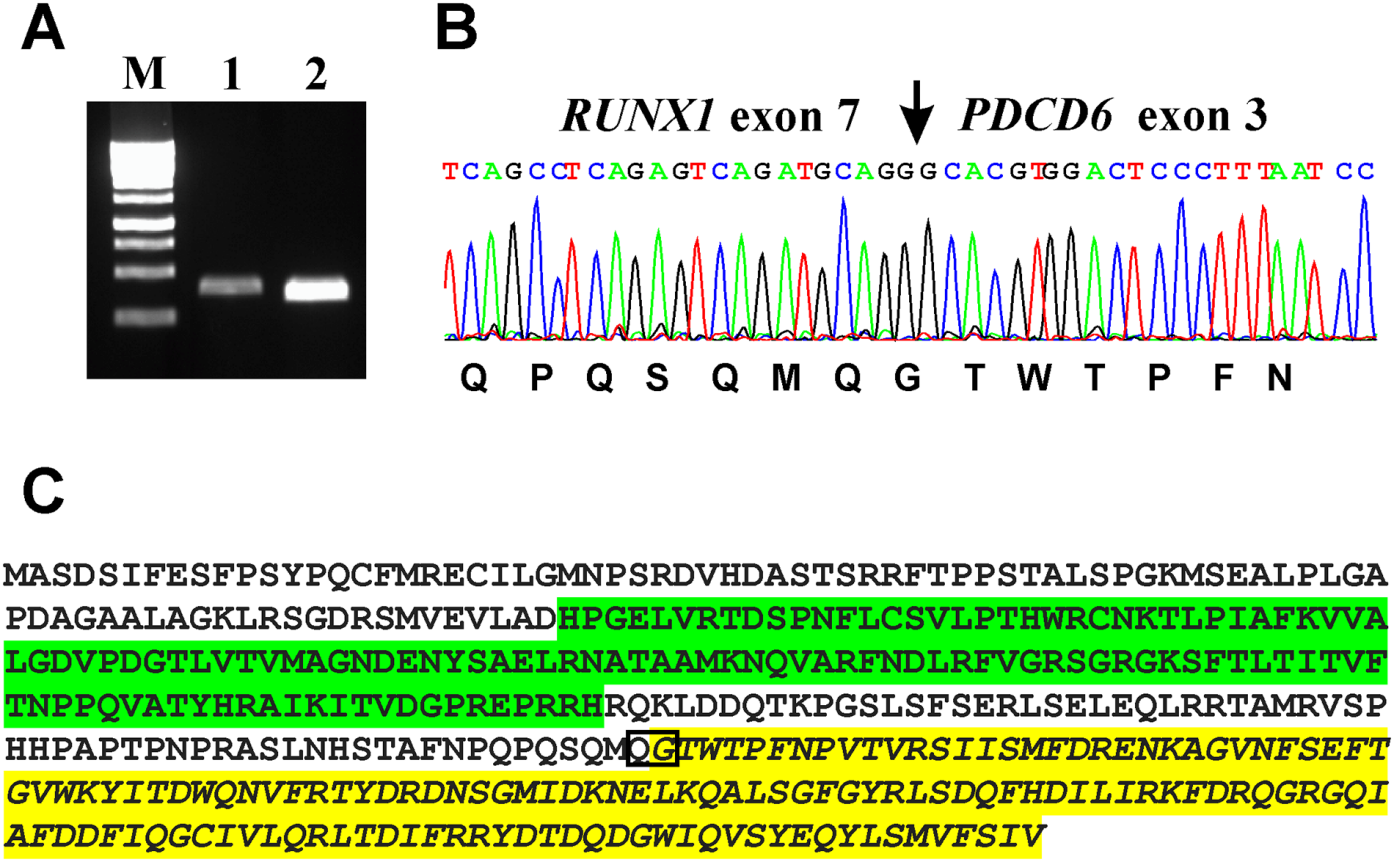
Molecular genetic analysis of bone marrow from a patient with CLL and secondary MDS. (A) Gel electrophoresis showing the amplified *RUNX1-PDCD6* cDNA fragments. M, GeneRuler 1 Kb DNA ladder (Thermo Scientific). Lane 1, amplification using the primer set RUNX1-809N-F1/PDCD6-354R1. Lane 2, amplification with the primer set RUNX1-852N-F1/PDCD6-373R1. (B) Partial sequence chromatogram of the cDNA fragment showing the fusion (arrow) of *RUNX1* and *PDCD6*. (C) The putative RUNX1-PDCD6 fusion protein. The Runt homology domain from RUNX1 is in green. The region with four EF-hand motives from PDCD6 is in yellow. The RUNX1-PDCD6 junction is in box.

PCR with the primer combinations RUNX1-809N-F1/PDCD6-354R1 and RUNX1-852N-F1/PDCD6-373R1 amplified 301 bp and 275 bp long cDNA fragments, respectively (Fig 3A). Direct sequencing of the PCR products verified the presence of *RUNX1-PDCD6* (Fig 3B). The fusion point was identical to that found by analysis of the RNA sequencing data using FusionCatcher (Fig 3B, Table 1). In the *RUNX1-PDCD6* transcript, exon 7 of *RUNX1* (nt 995 in sequence with accession number NM_001754version 4) was fused in frame to exon 3 of *PDCD6* (nt 264 in NM_013232 version 3) (Fig 3B and 3C).

**Fig 4:**
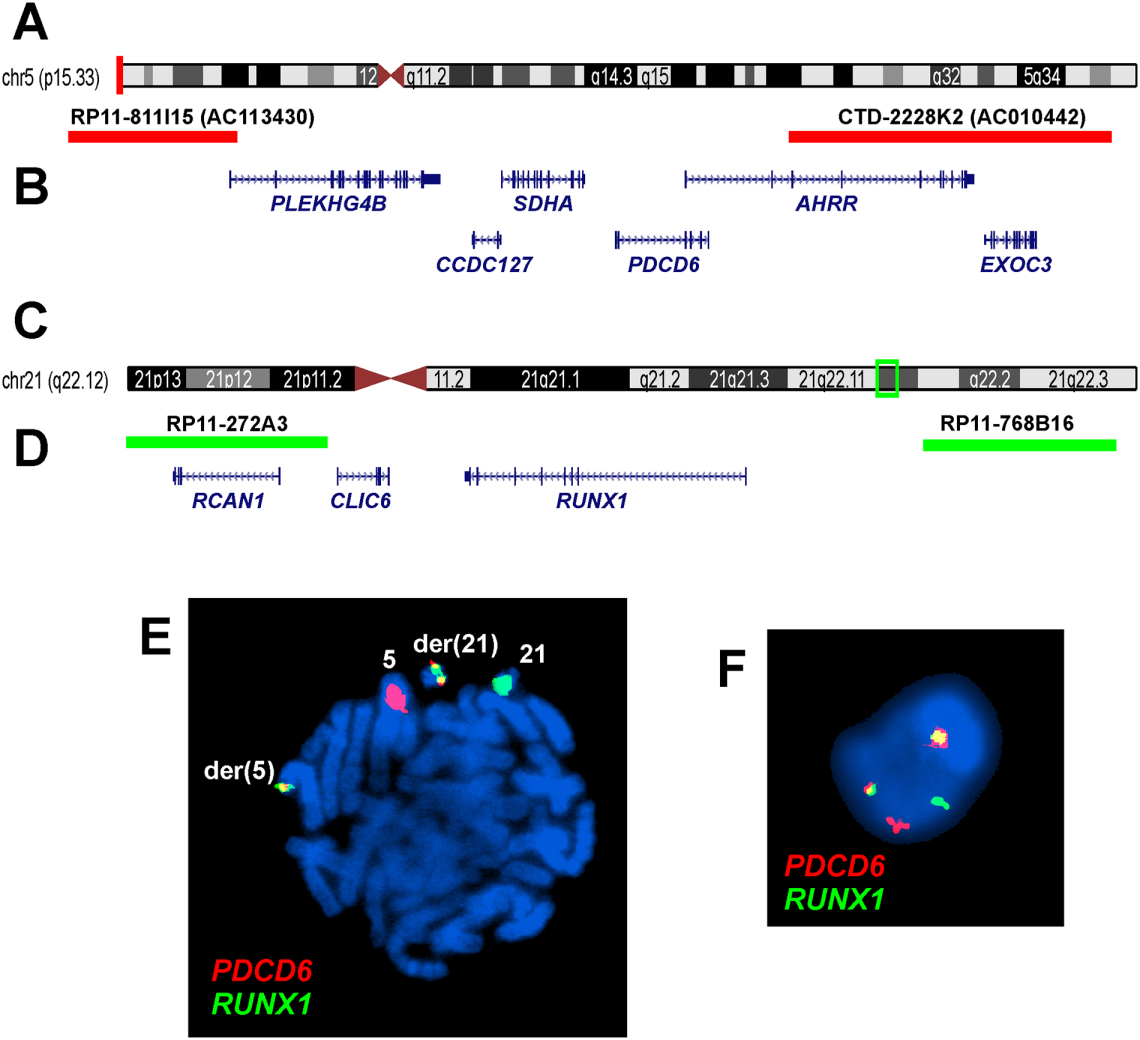
FISH analysis of bone marrow from a patient with CLL and secondary MDS using home-made break apart/double fusion PDCD6 and *RUNX1* probes. (A) Ideogram of chromosome 5 showing the mapping position of the *PDCD6* gene (vertical red line). (B) Diagram showing the FISH probe for *PDCD6*. Additional genes in this region are also shown. (C) Ideogram of chromosome 21 showing the mapping position of the *RUNX1* gene (green box). (D) Diagram showing the FISH probe for *RUNX1*. Additional genes in this region are also shown. (E) FISH on metaphase plate with the *PDCD6* (red signal) and *RUNX1* (green signal) probes showing a red signal on chromosome 5, a green signal on chromosome 21, and two yellow fusion signals, one on der(5) and one on der(21). (F) FISH on interphase nuclei showing a red *PDCD6* signal, a green *RUNX1* signal, and two yellow fusion signals.

Further FISH analyses with *PDCD6* (Fig 4A and 4B) and *RUNX1* (Fig 4C and 4D) home-made break apart/double fusion probes on metaphase spreads showed a red signal (*PDCD6*) on chromosome 5, a green signal on chromosome 21 (*RUNX1*), and two yellow fusion signals, one on der(5) and the other on der(21) (Fig 4E). Similar results, i.e., a green, a red, and two yellow fusion signals, were also seen in interphase nuclei (Fig 4F).

Reassessment of the G-banding preparations in light of the FISH and RNA sequencing data thus yielded the karyotype 46,XX,t(5;21)(p15;q22)[9]/46,XX[2].

## Discussion

We present here a case of CLL with secondary myelodysplasia in which the del(21)(q22) detected by G-banding analysis of leukemic cells turned out to correspond to a novel t(5;21)(p15;q22) leading to fusion of *RUNX1* from 21q22 with *PDCD6* from 5p15. The t(5;21)(p15;q22)/*RUNX1*-*PDCD6* was identified using a combination of molecular cytogenetics, RNA sequencing, and molecular genetic methodologies.

The *PDCD6* gene encodes a calcium-binding protein belonging to the penta-EF-hand family. It interacts with many other proteins, is associated with cell proliferation, and participates in T cell receptor-, Fas-, and glucocorticoid-induced programmed cell death [14–16]. *PDCD6* was also found to be a p53-responsive gene that induces apoptosis in response to DNA damage [17]. *PDCD6* is aberrantly expressed in several neoplasias and is important for tumor cell viability [16, 18–20].

Based on the karyotyping and FISH data, the *RUNX1-PDCD6* fusion gene should be generated on the der(5) chromosome. Based on the reference sequences NM_001754.4 and NP_001745.2 for *RUNX1* and NM_013232.3 and NP_037364.1 for *PDCD6*, *RUNX1-PDCD6* encodes a chimeric protein with 405 amino acid residues. It would contain the Runt homology domain from RUNX1 which is responsible for both heterodimerization with CBFB and DNA binding and four out of five EF-hand motives from PDCD6, each of which binds Ca^2+^ [15, 21]. The function of this fusion protein and its cellular consequences leading to leukemia are unknown. However, one can assume that the RUNX1-PDCD6 fusion protein is an abnormal transcription factor in a manner similar to what has been seen with other RUNX fusion proteins.

According to the Mitelman Database of Chromosome Aberrations and Gene Fusions in Cancer (https://cgap.nci.nih.gov/Chromosomes/Mitelman), del(21)(q22) has been found in the abnormal karyotypes of 21 patients with AML and 5 patients with MDS. In two patients, a 79-year-old man with refractory anemia with excess of blasts and a 47-year-old woman with AML with maturation (FAB type M2), del(21)(q22) was seen as the sole anomaly. In two others, a 61-year-old woman with AML without maturation (FAB type M1) and a 66-year-old woman with AML with maturation (FAB type M2), del(21)(q22) was found together with trisomy or tetrasomy 8, respectively.

In our patient, the t(5;21)(p15;q22) translocation could be identified only when what had seemed like a del(21)(qq) in G-banded preparations was examined using FISH and RNA-sequencing directed at finding out what lay behind the 21q-. Thus, at least some of the above-mentioned patients with del(21)(q22) in their karyotype might actually carry a t(5;21)(p15;q22). Further investigation of cases with del(21q) is necessary to determine the frequency of t(5;21)(p15;q22)/*RUNX1-PDCD6* in hematologic malignancies.
